# An Intelligent Smart Plug with Shared Knowledge Capabilities

**DOI:** 10.3390/s18113961

**Published:** 2018-11-15

**Authors:** Luis Gomes, Filipe Sousa, Zita Vale

**Affiliations:** GECAD-Research Group on Intelligent Engineering and Computing for Advanced Innovation and Development, Polytechnic of Porto (P.PORTO), P-4200-465 Porto, Portugal; ffeso@isep.ipp.pt (F.S.); zav@isep.ipp.pt (Z.V.)

**Keywords:** consumption forecast, distributed optimization, shared knowledge, smart plugs, user interaction forecasts

## Abstract

The massive dissemination of smart devices in current markets provides innovative technologies that can be used in energy management systems. Particularly, smart plugs enable efficient remote monitoring and control capabilities of electrical resources at a low cost. However, smart plugs, besides their enabling capabilities, are not able to acquire and communicate information regarding the resource’s context. This paper proposes the EnAPlug, a new environmental awareness smart plug with knowledge capabilities concerning the context of where and how users utilize a controllable resource. This paper will focus on the abilities to learn and to share knowledge between different EnAPlugs. The EnAPlug is tested in two different case studies where user habits and consumption profiles are learned. A case study for distributed resource optimization is also shown, where a central heater is optimized according to the shared knowledge of five EnAPlugs.

## 1. Introduction

Today’s market is filled with smart devices for our homes, selling the promise of smart control and monitoring. However, what does this really mean? To implement a smart home, typically multiple devices are required with a central system (using a centralized software with, or without, hardware). By using single or independent devices the only thing we achieve is a remote control and monitoring. Despite this preliminary limitation, this market is growing fast and it is expected that by 2022 there will be a total of 216.9 million homes worldwide with at least one smart device [1].

One of the key smart devices available is the smart plug. The oldest news, tracked by the authors, that presents the concept of the smart plug, is from 2008 where Woods presented an ‘intelligent’ plug for energy savings and energy efficiency [2]. Smart plugs enable the retrofitting of electrical resources and provide us with basic functionalities, such as scheduling or the creation of rules and scenes. However, to achieve a complete futuristic smart home solution, similar to the one seen in Smart House (1999) movie, is almost mandatory to have a virtual assistant with voice control support—meaning, one more system is required. A big challenge for users is also the aggregation of multiple systems and platforms from various brands, a job that can easily be overwhelming to the ordinary user.

Besides the smart plugs available on the market, some scientific publications propose new approaches and/or solutions. In [3,4], the authors propose smart plugs using Zigbee as a communication protocol. A Bluetooth approach is proposed in [5]. In [6], a low-cost smart plug using IEEE 802.11 for wireless communication is proposed. A more evolved smart plug is proposed in [7], with the goal of detecting and act during on- and off-peak periods.

Smart plugs can be used in energy management systems as enablers, providing monitoring and control capabilities. Smart plugs without monitoring are usually not suitable for energy management because of the lack of information provided by the smart plug. The works presented in [8,9,10] are examples where smart plugs were used as actuators in energy management systems.

In [11], a Message Queuing Telemetry Transport (MQTT) smart plug is integrated into an energy management system for remote actuation. In [12], is proposed a smart plug dedicated for integration with energy management systems. A centralized energy management system is also proposed in [13]. In [14], smart plugs are used for resource classification, using their consumption profile.

The problem with smart plugs is that they are not very smart and all of them have a limitation regarding resource and user information. An energy management system is highly dependent on the data and information available about the building’s context. To be able to perform quality energy optimizations, the system must somehow understand the building’s context, otherwise, it could result in low-quality optimizations, putting in risk users’ comfort or even the building’s security. Under these circumstances, this paper motivation aims to solve the lack of information regarding smart plugs context, so that they can be efficiently used in energy management systems, and to show the promising results of having a context awareness smart plug.

This paper proposes an evolution of Environmental Awareness Smart Plug (EnAPlug) that is focused on the context. The previous version of EnAPlug was made using a microcontroller and worked as a passive system [15,16]. The proposed evolution is not only able to understand the controllable resource’s context but also to learn from the resource consumption history and from the interaction between users and resources. The proposed EnAPlug has an agent-based architecture using a cooperative approach, where the knowledge learned can be shared between its peers to provide a decentralized resource optimization. The proposed methodology of shared knowledge is presented and is the main innovation of this paper.

Moreover, the paper presents the EnAPlug architecture and knowledge capabilities. Two physical installations of EnAPlugs are used as case studies: one in a refrigerator; and a second in a desk lamp. In addition, multiple artificial neural network configurations were tested and evaluated to provide knowledge to each EnAPlug. Finally, a distributed optimization scenario is presented using the shared knowledge EnAPlug capability.

After this first introductory section, a market survey is presented in Section 2. In Section 3, the proposed EnAPlug architecture is detailed, focusing on the shared knowledge and distributed optimization capabilities. Section 4 shows the learning results using two controllable resources. After that, Section 5 presents the outcome of the distributed optimization using the learning results. Finally, the discussion and main conclusions are presented in Section 6.

## 2. Market Available Smart Plugs

Smart plugs are easily found in current markets and their dissemination in people homes is a reality [1]. The first news regarding smart plugs appeared in 2008, discussing devices that can be remotely controlled and operated [2]. Since then, smart plugs have seen trivial evolution, and the base operational concept remains the same.

### 2.1. Smart Plugs Survey

The smart plugs market survey was conducted focusing only smart plugs with energy monitoring. The reason behind this choice is because smart plugs without energy monitoring capabilities cannot be used as unique elements in energy management systems, demanding external energy monitoring systems. The information presented in Table 1 was collected using official websites, datasheets, and manuals. If a certain functionality was not specified in any of those sources of information, it was assumed that the smart plug did not have such functionality. The price column was completed considering prices of official website and Amazon websites. The estimated price of EnAPlug only considers the prototype stage where is used a Mean Well DR-15-5 5 V power supply and a Circuitor CVM-1D energy analyzer, that represent more than 60% of the price. For a commercial version, these two components should be replaced or eliminated.

It is worth noting that Table 1 excludes not only smart plugs without monitoring functions but also smart plugs with monitoring whose functionalities and capabilities are not well described (i.e., when a significant lack of information was identified, the smart plug was not included in Table 1).

### 2.2. Problems and Limitations

The conventional smart plugs presented in Table 1 are not truly smart in any aspect. For instance, the smart plugs are enablers for retrofitting electrical resources but do not possess any smartness in their core. The authors could not find a standard definition of smart plugs, but the survey indicates the following definition: a smart plug is an electronic device for electrical outlets installation that can control an electric resource and is able to communicate, also, it may, or may not, have the ability of energy monitoring.

The application of smart plugs enables the concept of smart homes. However, a smart plug is only an enabler while the smartness and intelligence are in the smart home management system. If smart plugs were truly smart and intelligent, new business models, opportunities and features could appear on the market and in the open source community.

Nowadays, the clear disadvantage in the smart plug market is the lack of standardization or the implementation of standards in the smart plugs. Standards like Z-Wave or NEST were developed for home applications but are not the market standard for these products. This problem can affect the users and creates friction in the usage of smart plugs with third-party smart homes or energy management systems.

The use of multiple communication protocols, such as Z-Wave or Zigbee, demands the installation of hubs that aggregate all the communications and work as an interface between Wi-Fi or Ethernet network to the protocol used by the smart plugs. It is not unusual to have more than one hub in homes, and this can be a problem for users, representing an increase in equipment and in energy consumption. The application of Wi-Fi in smart plugs, although is more convenient, also raises other concerns regarding smart plug’s energy consumption.

The last limitation that the authors want to point out, concerns the quality of information measured by the current smart plugs. The values and parameters measured, if any, are overall related to the electrical resource consumption giving few or no information regarding the resource context. The inability to provide sufficient and quality information to a smart home or energy management system will limit the system actuation.

## 3. Proposed Architecture

**Figure 1:**
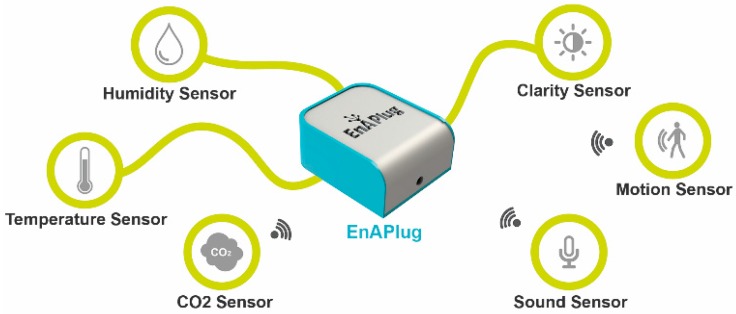
EnAPlug sensors integration.

Facing the limitations of current smart plugs, available in the market, the authors propose an improved EnAPlug. The proposed improved EnAPlug differentiates itself from others because of its ability to understand the context of the load and its ability to share knowledge. While the majority of conventional smart plugs only provide consumption values, EnAPlug can collect more data that can be transformed into knowledge. EnAPlug has a plug and play feature to integrate multiple sensors, as shown in Figure 1. This enables the measurement of parameters that directly or indirectly have an impact in the usage or operation of the electrical resource that EnAPlug controls.

The sensors of each EnAPlug should be chosen after the controllable resource is known. If the control will be done in a lamp, EnAPlug could have a movement sensor, a room luminosity sensor, and a noise sensor. These sensors will give a clear view about how and when the lamp is used.

The allocation of sensors in each EnAPlug should be thought out for each application. A sensor should be installed if the measured value has a direct or indirect connection in when or how the controllable resource will be used. The correct choice of sensors will increase the awareness capability of EnAPlug.

The proposed EnAPlug uses a multi-agent system (MAS) approach, that according to [17] should provide reactivity, pro-activeness and social abilities. These abilities allow the MAS to react to changes happening in their environment, to have pro-activity to achieve their own goals and to be social with other agents using direct communications to negotiate, collaborate or cooperate. The use of MAS for energy management systems functionalities is not a novelty feature, some examples can be seen in [18,19,20].

The proposed architecture implementation of an EnAPlug can be done using single-board computers (SBC). Currently, there are multiple low-cost SBC small enough to be integrated into the smart plug. Examples of low-cost and small SBC, with integrated Wi-Fi, are Raspberry Pi Zero, Orange Pi Zero, and NanoPi NEO Core2. This paper will focus on the software architecture, for the complementary hardware—sensors, actuators, and energy analyzer—please refer to [15,16].

**Figure 2:**
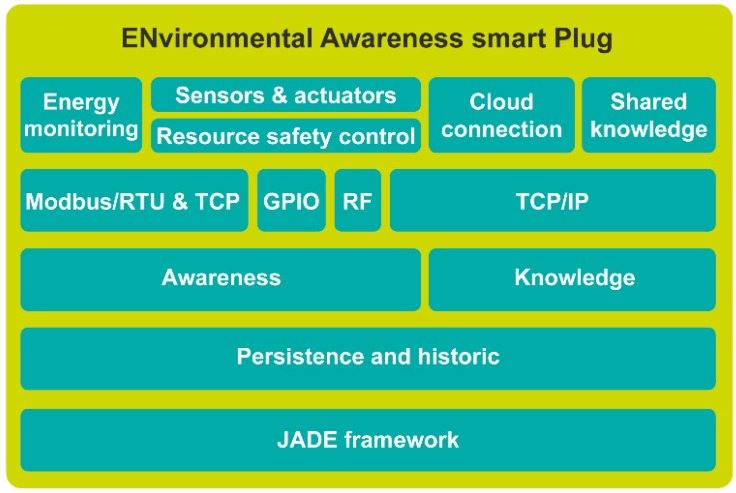
EnAPlug architecture.

The internal architecture of EnAPlug is shown in Figure 2. The base of EnAPlug is (Java Agent Development) JADE framework [21]. This framework enables a faster MAS development by providing FIPA compliance [22] and useful agents such as the Directory Facilitator that provides yellow pages to the MAS. Other frameworks can be used for this layer without compromising the proposed architecture.

On top of the JADE framework is the persistence and historic layer that stores all the information read and produced by the agent. For this layer, the use of SBC brings clear limitations in the storage size. Therefore, the authors recommend the connection between the persistence and historic layer to a cloud storage server. This will enable the agent to collect and store, and then send it to an external server, freeing storage space in the SBC.

The awareness layer is responsible for data acquisition and resource control. Therefore, has on its top four possible communications: Modbus/RTU & TCP, General Purpose Input/Output (GPIO), Radiofrequency (RF), and TCP/IP. The use of Modbus/RTU & TCP enables an easy communication between the SBC and energy analyzers—that usually possess a Modbus/RTU interface for remote access. Modbus/RTU & TCP can also be used for communication with sensors and actuators because the majority of SBC provide GPIOs, they can be used for sensor connection. The radiofrequency layer provides a software interface between the agent and RF modules, such as 433Mhz RF or NRF24L01 modules. This communication is for sensors and actuators that are not wired connected to EnAPlug, providing a wireless interface for small sensors and actuators that do not possess a Wi-Fi communication. The TCP/IP layer provides Wi-Fi and/or Ethernet communication between the agent and the world. It can be used for sensors and actuators on top of the awareness layer or for remote connections on top of the knowledge layer.

The resource safety control layer is placed below the sensors and actuators layer to prevent an unsafe and dangerous situation in the controlled resource. This layer has the ability to overwrite control signals to prevent damage in the controlled resource or prevent dangerous or unsafe situations to the resource context. For instance, if EnAPlug is connected to an air-conditioner located in a server room, its control must be done carefully to prevent damage in the servers, EnAPlug should have a temperature sensor to measure the room’s temperature, and the resource safety control will only enable control signals when the temperature is below a certain value, otherwise, the signal should the overwrite to prevent temperature increasing. The resource safety control layer can be parameterized to send user warning and alerts when dangerous situations are detected. This layer is one critical aspect in EnAPlug and takes advantage of its context awareness ability.

The knowledge layer gives meaning to the information of the awareness layer by creating knowledge using deep learning. The training of deep learning algorithms can be a huge task for SBC. Therefore, the training process of deep learning algorithms can be performed in the cloud, while the trained model can be used inside EnAPlug’s SBC. The Cloud connection enables the interaction between EnAPlug and a cloud where deep learning algorithms can be trained using EnAPlug’s data.

The shared knowledge layer is described in detail in the next Section 3.1. This layer enables the share of knowledge directly between EnAPlugs to build a shared knowledge world where each EnAPlug contributes with their knowledge.

### 3.1. Shared Knowledge

The shared knowledge layer is one of the most important layers in EnAPlug’s architecture. The ability to learn and share its knowledge is the main feature of EnAPlug and brings a new set of possibilities to its application.

The control and monitoring of electrical resources are important enablers but is possible to turn smart plugs in devices with knowledge regarding the usage and operation of a controllable resource. For this to be possible, the proposed architecture presents a knowledge layer that can use remote deep learning algorithm for learning using the data of the awareness layer (Figure 2). The knowledge layer brings knowledge to EnAPlug, while the shared knowledge layer brings the ability to share this knowledge with other EnAPlugs.

Knowledge layer is responsible to give meaning to data. The use of this layer can be vast and different from each EnAPlug application, but for this paper it is considered that the Knowledge layer should have answers to the following two questions:

- The resource will be used by the user in the next hour?
- The resource will have electrical consumption in the next hour?

Each EnAPlug should be able to answer those two questions in thirty minutes ahead. In some cases, such as televisions and heaters, the answers of both questions can be the same—both positive or negative—but in some cases, such as water heaters and refrigerators, the answers can differ.

EnAPlug should also understand its surroundings and context besides the knowledge provided by the knowledge layer. Therefore, each EnAPlug must be able to answer, at any time, to the following two questions:

- The resource is being used?
- The resource is consuming electricity?

**Figure 3:**
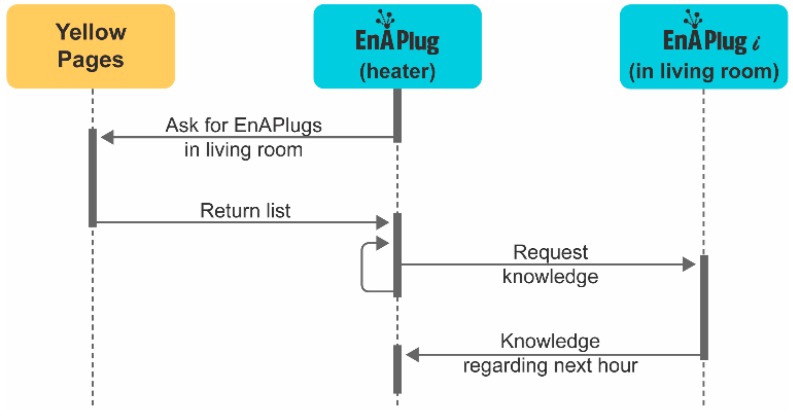
Shared Knowledge procedure.

The shared knowledge feature enables EnAPlugs to interact with each other’s to build a complete movement and presence mind map of the building where they are installed. An EnAPlug, can, at any time, quiz others regarding their knowledge. Figure 3 shows the communication sequence regarding knowledge acquisition.

In Figure 3, is presented an example where a heater’s EnAPlug, located in living room, wants to optimize its consumption according to the usage of the living room. Therefore, it must ask every EnAPlug in the living room if its resource is in used and if the resource will be used during the next hour. In order to do that, the heater’s EnAPlug will inquire the yellow pages of the MAS, provided by the Directory Facilitator agent of JADE framework, to request all the agents located in the living room. Then, for each agent in the living room, the heater’s EnAPlug will inquire the agent knowledge.

**Figure 4:**
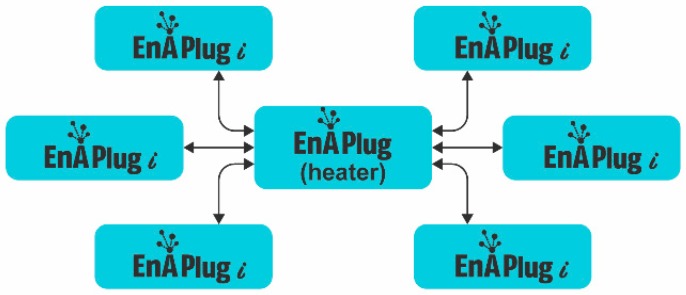
EnAPlugs connection for shared knowledge.

The shared knowledge layer enables the collaboration between EnAPlugs, all of them are connected and can benefit from each other’s, as seen in Figure 4. Like in a society, the combination of knowledge leverages the overall knowledge and can improve resource management. In the previous example, regarding the heater’s EnAPlug, the heater should management its consumption using the knowledge of every resource located inside the living room.

All EnAPlugs contribute to an overall shared knowledge that is available to all in a distributed and decentralized way, avoiding the centralization of knowledge. However, energy and resource centralized management solutions can be built on top of EnAPlug to use their context capabilities.

The questions that EnAPlug should answer can expand to include new ones. The proposed four questions are used in our case study, but more can be included.

The shared knowledge layer is built upon the knowledge layer, while the knowledge layer is built upon the data from the sensors & actuators layer. Therefore, the application of sensors must provide enough data to the knowledge layer. The use of the right sensors in the EnAPlug is the most important aspect, without the right sensors, the ability to transform data into knowledge and then share it with the others is not possible.

### 3.2. Distributed Optimization

EnAPlug can be integrated into an energy management system for centralized resource optimization. However, EnAPlugs are able to run distributed optimizations, where each EnAPlug optimizes their own resource consumption according to the shared knowledge provided by other EnAPlugs.

The shared knowledge is very important for distributed optimizations. EnAPlug can use the shared knowledge of all EnAPlugs located in the same room to identify if there will be persons in the room during the next hour.

To use the shared knowledge for distributed optimization, Equation (1) is used. Where *A* is the EnAPlug’s answer—there will, or will not, be users/consumption during the next hour. The aggregation of knowledge is calculated using Equations (2) and (3). To know if there will be users/consumption in the room, at least one EnAPlug should respond affirmatively. However, to know if there will not be users/consumption in the room, all the EnAPlugs should respond negatively.

Equation (2) calculates the probability of a negative room answer. Where *E*_0*i*_ is the EnAPlug *i* probability of not having users/consumption. Equation (3) calculates the probability of an affirmative answer—adaptation from the principle of inclusion and exclusion for probability [23]. Where *E*_1*i*_ is the EnAPlug *i* probability of having users/consumption.

Equation (4) calculates the EnAPlug probability of having (*E*_1_) or not having ($E_{0}$) users/consumption. For this, the answer is given by EnAPlug, represented by *A*, and the accuracy of its forecast, represented by γ, are used. For instance, if we have an EnAPlug, with an accuracy of 80%, giving a negative answer, then *E*_1_ = 20% while *E*_0_ = 80%. This is only true because they are binary answers.

(1) $$A_{room}=\{\begin{array}{c} 0,ifA_{1}+A_{2}+\cdots +A_{k}=0\\ 1,ifA_{1}+A_{2}+\cdots +A_{k}>0 \end{array}$$

(2) $$\beta_{0_{room}}=\overset{n}{\underset{i=0}{\prod }}E_{0_{i}}$$

(3) $$\beta_{1_{room}}=\overset{n}{\underset{k=1}{\sum }}\left({\left(-1\right)}^{k-1}\underset{\begin{array}{c} i_{1},i_{2},\ldots ,i_{k}:\\ 1\leq i_{1}<i_{2}<\cdots <i_{k}\leq n \end{array}}{\sum }P\left(E_{1_{i_{1}}}\cap E_{1_{i_{2}}}\cap \ldots \cap E_{1_{i_{k}}}\right)\right)$$

(4) $$E_{a_{i}}={\left(1-\gamma \right)}^{\left|a-A\right|}\gamma^{\left|1-a-A\right|}$$

## 4. Learning Results

The deep learning mechanism used in this paper case studies was executed in EnAPlug using a trained model that was trained in the cloud—to avoid the low-processing of SBC. It was used Deep Learning for Java (DL4J) library to run artificial neural networks (ANN) [24]. The ANN chosen were the Long Short-Term Memory Units (LSTMs) because of their ability to continue learning [25,26,27]. It was used backpropagation networks, where the weights are initialized using the equation proposed in [28]. Gradients updates are calculated using Nesterov momentum. A threshold of 0.5 is applied to truncate values, as it was proposed in [29].

To avoid underfitting and overfitting, it was used the early stopping functionality available in DL4J [30]. The early stopping analyzes an ANN using a time window and a maximum number of epochs, and it is able to calculate the equilibrium point where the ANN is not underfitting or overfitting, giving the best epoch number for training.

The dataset used for ANN uses real data collected using two physically installed EnAPlugs in our research center. A total of 106 ANN were designed, trained and evaluated. For this paper, only the best configurations were included. For each configuration is presented the two best sub-configurations. Where a configuration differs from each other by their inputs and outputs, while sub-configuration only varies according to their internal composition—hidden layers and number of neurons.

### 4.1. Refrigerator

It was installed an EnAPlug in a refrigerator containing an energy analyzer, outside temperature sensor, inside temperature sensor, inside a humidity sensor and a door sensor that detects the opening of the door. The EnAPlug sensors were installed to gather enough context data from the refrigerator usage. Data from this installation can be found in [16].

The inside temperature sensor is used by the resource safety control layer to prevent damage in the refrigerator goods. If inside temperature exceeds more than 7 °C, the safety control will turn on the refrigerator and overwrite the resource remote control signals.

At this point, EnAPlug controls the refrigerator as an all and for this reason, where the power is cut, the motor and inside light stop working. A new actuator mechanism can handle the cut of the motor only, but it would require changes inside the refrigerator.

The knowledge layer on this EnAPlug uses artificial neural networks (ANN). The ANN was tested using several inputs and training sets. In the end, were used two different ANN, one to forecast if the refrigerator will be used by the users in the next hour, and other to forecast if the refrigerator will have consumption in the next hour.

The light consumption of the refrigerator—when the door is open—is not considered as relevant consumption. Therefore, the forecast for refrigerator consumption will only forecast the motor consumption and not the 20 W light consumption.

Table 2 shows the seven best ANNs to answer the shared knowledge questions (Section 3.1). The configurations differ by their input and output parameters. The configurations numbered as 1.1.*x* answer the first question regarding the usage of the resource in the next hour, while the configurations numbered as 1.2.*x* answer the second question regarding the consumption of the resource in the next hour. The configurations are executed in *t*-30 min where *t* corresponds to the hour that is forecast.

Has said before, the number of epochs where previously calculated using the early stopping functionality of DL4J to prevent the under and overfitting of the network.

The results show that Configuration 1.1.2 is the best option to forecast the usage of the refrigerator. This algorithm forecasts, with a 91% accuracy, if the door of the refrigerator will be open during the next hour—meaning that at least one user will be at the kitchen in the next hour. Table 3 shows the detailed results for the Configuration 1.1.2 with 20 nodes in the hidden layer and using a 3496 epochs training. As can be seen, the algorithm was able to forecast 95.8% of the time if the door will not be open, representing an 8.9% improvement above the chance. The forecast for hours where the door will be open had a 60.9% accuracy, representing an improvement of 407.3% above chance. Meaning that the ANN, with an accuracy of 60.9% for hours that the door will be opened, is still four times better than the chance of that scenario really happen.

To answers the question of whether the EnAPlug resource will consume electrical energy during the next hour, Configuration 1.2.2 and 1.2.4 are the best options. The algorithms differ from their outputs, Configuration 1.2.2 has two possible results while 1.2.4 has four possible outcomes. Table 4 shows the detailed results of Configuration 1.2.2 that can forecast if the EnAPlug resource will, or will not, consume electrical energy in the following hour. Configuration 1.2.2 uses an ANN with 10 hidden nodes in a single hidden layer with an 1136 epochs training. With an 88.4% overall accuracy, Configuration 1.2.2 brings clear advantages, between 75.6% and 78%, above the change.

Facing the good results of Configuration 1.2.2; Configuration 1.2.3 and 1.2.4 were designed using four possible outcomes. Instead of forecasting only if the refrigerator motor will consume, these new algorithms try to better understand when this consumption will happen. The possible outputs of these algorithms are: there will not be any consumption during the forecasted hour; there will be consumption only in the first half an hour; there will be consumption only in the last half an hour; or there will be consumption during the entire hour.

Table 5 shows the detailed results of Configuration 1.2.4 using a network with 5 hidden nodes and a 1088 epochs training. The results show a significant improvement in the above chance values. The configuration can, with an accuracy between 58.1% and 91.5%, forecast if the refrigerator motor will run in the first half an hour, in the last half an hour, in the full hour or if it will not run at all. With an overall accuracy of 81.6% using four possible outcomes, the Configuration 1.2.4 is a good option to forecast if the refrigerator will, or will not, consume electrical energy in the following hour period.

### 4.2. Desk Lamp

In this case study, the desk lamp is used for desk works, and it should be turned on every time a person is working on the desk. This way, the electrical consumption is directly connected to the presence of the user. By considering this, the forecast questions of Section 3.1 can be answered using a unique configuration. The ANN that forecasts the user presence is also used for the consumption forecast. This is only possible because this resource is dependent on user usage, and only has consumption if the user is there.

The desk lamp’s EnAPlug was integrated with a luminosity sensor, a temperature sensor, and a presence sensor. The presence sensor itself is the combination of a passive infrared sensor (PIR) that is a movement sensor, a keyboard sensor, and a mouse sensor. The keyboard and mouse sensors are purely software installed on the desktop computer. It is the combination of those three sensors that provide the information of user presence. Table 6 shows the three best configurations used for the forecast. The ANN was built in DL4J using the early stopping functionality.

Table 7 analyzes, in detail, the results of Configuration 2.1 using 20 nodes in the hidden layer and a 1043 epochs training. This ANN configuration achieved great numbers above change. For instance, there is a change probability of 24.4% for the desk to be used, but using the ANN we achieve an 81.5% accuracy, resulting in 234.2% above change.

Each configuration of Table 2 and Table 6 were executed with several hidden layer configurations, Table 8 shows the two best sub-configurations results. Configuration 1.1.2 was the best configuration to forecast refrigerator usage, while Configuration 1.2.2 had the best result for consumption forecast. The results of Table 8 show that the configuration with the best result for the desk lamp was Configuration 2.1 with 92.0% accuracy, while Configurations 2.2 and 2.3 were very close achieving results of 91.80% accuracy.

## 5. Distributed Optimization Results

**Figure 5:**
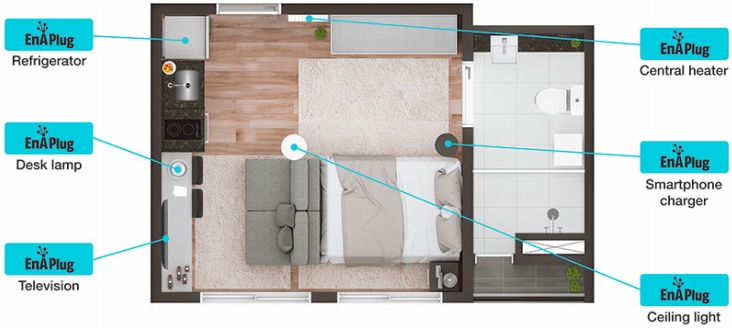
Apartment EnAPlugs distribution.

As previously said, the main contribution and disruption between EnAPlug and market smart plugs is the ability to collaborate and share knowledge between them. This case study considers a T0 apartment where six EnAPlugs are deployed, according to Figure 5:

- *Refrigerator*—with a 91% accuracy ANN (Configuration 1.1.2);
- *Desk lamp*—with a 92% accuracy ANN (Configuration 2.1);
- *Television*—with a 90% accuracy ANN;
- *Smartphone charger*—with an 87% accuracy ANN;
- *Ceiling light*—with a 92% accuracy ANN;
- *Central heater*—with distributed optimization.

**Figure 6:**
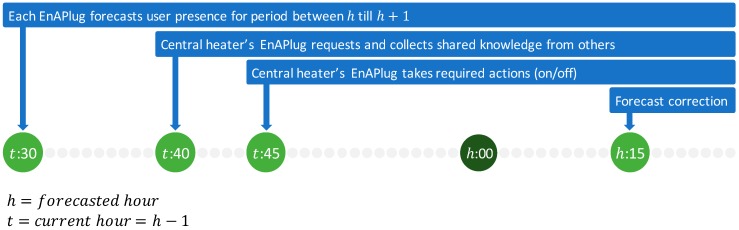
EnAPlug’s distributed optimization timeline.

The distributed optimization, provided by EnAPlug, uses the shared knowledge capability to combine knowledge from different smart plugs in order to optimize the consumption of a resource. In this case study, is used a central heater. Figure 6 shows the timeline of this optimization process. The hour that the optimization will run is defined by h, while t defines the hour before h. Thirty minutes before, the hour beings, each EnAPlug executes their forecasts. After ten minutes, the central heater’s EnAPlug uses the shared knowledge capability to request, to each EnAPlug, their knowledge, and five minutes later, the central heater’s EnAPlug will control its resource–turning it on if there will be users inside the house or turn it off otherwise. In this case study, the central heater is turned on when a forecast has an equal or higher probability of 90% for having users. At h:15, the central heater’s EnAPlug requests the share knowledge once again, but this time, to ask if any EnAPlug was used in the last fifteen minutes. The new request will detect, and correct false positives or negatives resulted in the forecast action.

The use of forecast to control central heaters could improve users conform by eliminating the starting time—the time between the heater is turned on and the time that the house starts increasing its temperature. The heater can, in this way, be turned on ahead in time, leaving the house heated before the user’s arrival. This case study uses the indicative ahead control time of fifteen minutes. However, the ahead control time should be directly related to the starting time of the heater, varying according to each heater.

Half an hour, previously the optimized hour, each EnAPlug uses its forecasts methods to forecast if the user will use its controllable resource and if the controllable resource will consume energy. This case study will only use the user usage forecast, unlike the previous case studies where both forecasts were analyzed in detail. Table 9 shows the forecasts of each EnAPlug in this case study, and the result of Equation (4), for $E_{1}$.

When Equations (3) and (4) are applied to the results of Table 9 the central heater’s EnAPlug gets the following values:

- the probability of not having users in the next hour (Equation (3)): **0.058%**
- the probability of having users in the next hour (Equation (4)): **99.938%**

With a 99.94% probability of being someone home during the next hour, the central heater’s EnAPlug turns on the heat of the house before the user’s arrival. This ahead action is also valid for hours where the user will not be there—meaning, if the user is at home, but the central heater’s EnAPlug receives the information that the user will not be there during the next hour, the heater will be turned off before the end of the hour to avoid overheating in the last minutes of the hour.

The shared knowledge capability allows the combination of multiple knowledge, provided by different EnAPlugs. This combination improves the overall forecast by minimizing the associated error in the entire house. As seen, the individual certainty for $E_{1}$, in each EnAPlug, results in the lowest values when compared to the house certainty of 99.94%. The ability to share their knowledge improves the overall agent community knowledge in a local distributed system.

## 6. Discussion and Conclusions

The integration of smart plugs in energy management systems can serve as enablers for resource monitoring and control, enabling retrofitting in today’s homes. However, this integration has clear limitations regarding resource context information.

Contextual data can be read with sensors, and there are several approaches to where the sensors should be placed and how the information should be collected. One possible approach is to use market sensors for smart homes, this will enable the understanding of context regarding resources and houses/buildings—on the basis that they are well placed. However, it will require the integration of all these sensors and the development of a system that can read the sensors data and act on smart plugs. Another possible approach is to use room sensors and remove the share knowledge ability. However, this will not provide individual resource information and will generate only one forecast result. The use of share knowledge allows the reduction of the forecast error inside a room/house/building. The approach of EnAPlugs do not demand a cloud system and all the process can be executed within the plugs, even when a cloud server is used for deep learning training, as seen in this paper, the real-time data and forecasts, provided by the trained model, will not be sent to the cloud, minimizing the user data exposure.

This paper proposes an intelligent smart plug with shared knowledge capabilities. The proposed agent-based smart plug, named EnAPlug, is a context awareness smart plug that can learn some aspects related to the controllable resource. The real-time context measurements and the learned knowledge can be shared between other agents in a cooperative and open way, without any restriction—taking the fact that all agents must belong to the same system.

The presented case study demonstrated the learning ability using as controllable resources: a refrigerator, and a desk lamp. The learning methodology uses long short-term memory recurrent neural networks. The case study also demonstrates the benefits of the shared knowledge functionality, where the knowledge of five EnAPlugs are used to optimize the central heater consumption according to users’ presence. The results show that the architecture of EnAPlug brings clear advantages for energy management systems, mainly because of its context awareness capabilities.

## Figures and Tables

**Table 1:** Overview of Market Smart Plugs and EnAPlug.

Smart Plug	Wireless Protocol	Max. Power (W)	Price (EUR)	Hub (required)	Amazon Alexa	Apple HomeKit	Google Assistant	Nest	Home Assistant	IFTTT	Agenda/Schedule	Rules	Scenes	External Sensors
*ANKUOO NEO PRO SW3101M*	IEEE 802.11b/g/n	3680 *	32.15	×	×	×	×	×	✓	×	✓	×	✓	×
*BroadLink Smart plug SP3*	IEEE 802.11b/g/n	3600	24.90	×	✓	×	×	×	✓	×	✓	✓	✓	×
*D-Link DSP-W215*	IEEE 802.11n	1800	36.12	×	✓	×	✓	×	✓	✓	✓	✓	✓	×
*EDIMAX SP-2101W V2*	IEEE 802.11b/g/n	3680 *	56.82	×	✓	×	×	×	✓	×	✓	×	×	×
*Elgato-Eve Energy*	BLE	2500 *	49.99	✓	×	✓	×	×	×	×	✓	✓	✓	×
*Fibaro Wall Plug*	Z-Wave	2500	58.78	✓	×	×	×	×	✓	×	✓	✓	✓	×
*Fibaro Wall Plug (BLE)*	BLE	2500	75.13	✓	×	✓	×	×	✓	×	✓	✓	✓	×
*iDevices SWITCH*	IEEE 802.11b/g/n and BLE	1800	23.74	×	✓	✓	✓	×	×	×	✓	×	×	×
*iSocket Environment Pro*	GSM	4000	143.20	×	×	×	×	×	×	×	✓	×	×	✓
*Koogeek P1 Plug*	IEEE 802.11b/g/n	1800	22.07	×	✓	✓	✓	×	×	×	✓	×	✓	×
*ORVIBO Smart WiFi Meter Plug 31*	IEEE 802.11b/g/n	1800	21.20	×	✓	×	×	×	×	×	✓	✓	✓	×
*Revogi Smart Meter Plug*	BLE	4000	20.31	×	×	×	×	×	×	×	✓	✓	×	×
*Sonoff S31 Smart Socket*	IEEE 802.11b/g/n	3520	14.54	×	✓	×	✓	✓	✓	✓	✓	✓	✓	×
*TP-Link HS110*	IEEE 802.11b/g/n	1800	32.90	×	✓	×	✓	✓	✓	✓	✓	×	✓	×
*TP-Link RE370K*	IEEE 802.11ac/n/g/b	3840	62.48	×	✓	×	✓	×	×	×	✓	×	✓	×
*Wemo Insight Smart Plug*	IEEE 802.11/n	1800	30.91	×	✓	×	✓	✓	✓	✓	✓	✓	✓	×
*Xiaomi Mi Smart Socket Plug*	IEEE 802.11b/g/n	2200	18.37	×	×	×	✓	×	✓	×	✓	×	×	×
*Proposed EnAPlug*	IEEE 802.11b/g/n	7360 **	~130	×	×	×	×	×	✓	×	✓	✓	✓	✓

* The maximum power changes according to the plug type; ** When installed in an electrical board, otherwise is limited to 16 A of a standard type-F plug.

**Table 2:** ANN configurations—for refrigerator EnAPlug.

**Configuration 1.1.1**	
**Inputs**	**Output**
[0–23]—hour of the day [1–7]—day of the week [0–1]—if the door was opened in the previous hour	[0–1]—if the user will open, or not, the refrigerator
**Configuration 1.1.2**	
**Inputs**	**Output**
[0–23]—hour of the day [0–1]—if week or weekend [0–1]—if the door was opened in the previous hour	[0–1]—if the user will open, or not, the refrigerator
**Configuration 1.1.3**	
**Inputs**	**Output**
[0–23]—hour of the day [0–1]—if week or weekend	[0–1]—if the user will open, or not, the refrigerator
**Configuration 1.2.1**	
**Inputs**	**Output**
kWh—consumption in last 30 min °C—refrigerator’s temperature °C—room temperature	[0–1]—if refrigerator motor will run
**Configuration 1.2.2**	
**Inputs**	**Output**
°C—refrigerator’s temperature °C—room temperature	[0–1]—if refrigerator motor will run
**Configuration 1.2.3**	
**Inputs**	**Output**
[0–23]—hour of the day [0–1]—if week or weekend [0–1]—if the door was opened in the last 15 min kWh—consumption in last 15 min kWh—consumptions in second to last 15 min °C—refrigerator temperature %—refrigerator humidity °C—room temperature	[0–3]—if refrigerator motor runs in the first half hour or/and in the second half hour (i.e., 0: not runs; 1: runs in the first half; 2: runs in the second half; 3 runs in first and second half)
**Configuration 1.2.4**	
**Inputs**	**Output**
[0–23]—hour of the day kWh—consumption in last 30 min °C—refrigerator temperature %—refrigerator humidity °C—room temperature	[0–3]—if refrigerator motor runs in the first half hour or/and in the second half hour (i.e., 0: not runs; 1: runs in the first half; 2: runs in the second half; 3 runs in first and second half)

**Table 3:** Refrigerator usage forecast—Configuration 1.1.2 with a single hidden layer with 20 neurons.

	Will Not Open	Will Open
*Positive*	413	42
*False Positive*	18	27
*Accuracy*	95.82%	60.87%
*Above chance*	8.89%	407.25%
*Total Accuracy*	91.00%	
*Total Precision*	81.93%	

**Table 4:** Consumption forecast—Configuration 1.2.2 with 10 hidden neurons.

	Will Not Consume	Will Consume
*Positive*	219	223
*False Positive*	26	32
*Accuracy*	89.39%	87.45%
*Above chance*	78.06%	75.60%
*Total Accuracy*	88.40%	
*Total Precision*	88.42%	

**Table 5:** Consumption forecast—Configuration 1.2.4 with five hidden neurons.

	Will Not Consume	Will Consume (First 30 min)	Will Consume (Last 30 min)	Will Consume (All Hour)
*Positive*	225	50	25	108
*False Positive*	21	22	18	31
*Accuracy*	91.46%	69.44%	58.14%	77.70%
*Above chance*	82.93%	460.04%	376.55%	205.90%
*Total Accuracy*	81.60%			
*Total Precision*	74.19%			

**Table 6:** Forecast configurations description—for desk lamp EnAPlug.

**Configuration 2.1**	
**Inputs**	**Output**
[0–23]—hour of the day [0–1]—if week or weekend lux—luminosity near the desk °C—temperature [0–1]—presence sensor in the last 30 min	[0–1]—if the user will, or not, use the desk, resulting in electrical consumption
**Configuration 2.2**	
**Inputs**	**Output**
[0–23]—hour of the day lux—luminosity near the desk °C—temperature	[0–1]—if the user will, or not, use the desk, resulting in electrical consumption
**Configuration 2.3**	
**Inputs**	**Output**
[0–23]—hour of the day [0–1]—if week or weekend lux—luminosity near the desk	[0–1]—if the user will, or not, use the desk, resulting in electrical consumption

**Table 7:** Desk lamp forecast—Configuration 2.1 with 20 hidden neurons.

	Will Not Be Used	Will Be Used
*Positive*	354	106
*False Positive*	16	24
*Accuracy*	95.68%	81.54%
*Above chance*	26.56%	234.17%
*Total Accuracy*	92.00%	
*Total Precision*	88.61%	

**Table 8:** ANN results.

	Hidden Neurons	Epochs	Dataset Size	Training Ratio	Test Ratio	Evaluation Dataset	Accuracy	Precision
*Config. 1.1.1*	10	2056	2500	80%	20%	500	89.80%	78.45%
*Config. 1.1.1*	3	3300	2500	80%	20%	500	89.00%	77.89%
*Config. 1.1.2*	20	3496	2500	80%	20%	500	91.00%	81.93%
*Config. 1.1.2*	30	1655	2500	80%	20%	500	91.00%	81.93%
*Config. 1.1.3*	10	2327	2500	80%	20%	500	89.58%	77.95%
*Config. 1.1.3*	2	48	2500	80%	20%	500	87.78%	74.34%
*Config. 1.2.1*	10	569	2500	80%	20%	500	86.80%	86.83%
*Config. 1.2.1*	50	780	2500	80%	20%	500	85.80%	85.89%
*Config. 1.2.2*	5	1208	2500	80%	20%	500	88.00%	88.08%
*Config. 1.2.2*	10	1136	2500	80%	20%	500	88.40%	88.42%
*Config. 1.2.3*	2	5657	2500	80%	20%	500	77.00%	72.81%
*Config. 1.2.3*	3	3244	2500	80%	20%	500	80.20%	75.31%
*Config. 1.2.4*	5	1088	2500	80%	20%	500	81.60%	74.19%
*Config. 1.2.4*	40	651	2500	80%	20%	500	80.20%	74.75%
*Configuration 2.1*	10	1438	2500	80%	20%	500	91.00%	88.74%
*Configuration 2.1*	20	1043	2500	80%	20%	500	92.00%	88.61%
*Configuration 2.2*	10	942	2500	80%	20%	500	91.40%	90.01%
*Configuration 2.2*	20	746	2500	80%	20%	500	91.80%	89.49%
*Configuration 2.3*	5	220	2500	80%	20%	500	89.20%	88.33%
*Configuration 2.3*	20	2057	2500	80%	20%	500	91.80%	89.49%

**Table 9:** EnAPlug’s results.

	Accuracy	Forecast Result	*E*_1_
*Refrigerator*	91%	There will be a user	91%
*Desk lamp*	92%	There will not be users	8%
*Television*	90%	There will be a user	90%
*Smartphone charger*	87%	There will not be users	13%
*Ceiling light*	92%	There will be a user	92%
